# Implications of the presence of yeasts in tracheobronchial secretions of critically ill intubated patients

**DOI:** 10.17179/excli2019-1631

**Published:** 2019-09-09

**Authors:** Elenice Gomes Ferreira, Fabrício Yatsuda, Marcio Pini, Isabele Carrilho Jarros, Flávia Franco Veiga, Admilton Gonçalves de Oliveira, Melyssa Negri, Terezinha Inez Estivalet Svidzinski

**Affiliations:** 1 Graduate Programme in Health Sciences, Universidade Estadual de Maringá (UEM), Maringá, PR, Brazil; 2 Department of Physiotherapy UniCesumar, Maringá, PR, Brazil; 3 PIC/UniCesumar/ICETI (Instituto Cesumar de Ciência, Tecnologia e Inovação); 4 Laboratory of Electron Microscopy and Microanalysis, Universidade Estadual de Londrina, Londrina, PR, Brazil; 5 Division of Medical Mycology, Teaching and Research Laboratory in Clinical Analyses, Department of Clinical Analysis of State University of Maringa, Avenida Colombo 5790, 87020-900 Maringá, PR, Brazil

**Keywords:** intubation, intratracheal, ventilators, mechanical, biofilms, Candida

## Abstract

The presence of some microorganisms in the respiratory tract is a known risk factor for the infection of air passages; however, it is not clear whether this holds true for *Candida* spp. Thus, our objective was to determine the frequency of yeast colonization in the tracheobronchial secretions of critically ill intubated patients and to assess the presence of these yeasts in the infra-cuff region of the endotracheal tube (ET). Patients aged 18 years or older who had been using an endotracheal tube for 48 hours were recruited. Tracheal secretions were collected; after extubation, the ETs were cut into two fragments in the infra-cuff region. One of these fragments was placed in a solution containing antibiotics and sent to the lab for culture and identification of yeasts. The remaining fragment was fixed and subjected to scanning electron microscopy (SEM). In total, 20 patients with an average age of 73.3 years (± 13.1) participated in this study. These patients remained under endotracheal intubation and invasive mechanical ventilation for an average of 6.4 (± 1.8) and 13.5 days (± 15), respectively. Of these patients, 45 % showed respiratory tract colonization by yeasts of the *Candida* genus, with *C. albicans* being the most frequently isolated species (66.7 %). Moreover, in almost 90 % of these patients, blastoconidia of the same yeast were found in the infra-cuff portion of the ET, as evidenced by SEM, strongly fixed on the ET surface. Yeasts isolated from both the infra-cuff region and the tracheobronchial secretions were susceptible to amphotericin B and fluconazole. In conclusion, our results show that the frequency of colonization by yeasts of the *Candida* genus in the tracheobronchial secretions of intubated patients within 48 hours is high, and that these species can also be found as a biofilm on the ET surface.

## Introduction

Candidemia or invasive candidiasis (IC) is one of the most common invasive fungal diseases, affecting a large number of people worldwide, with mortality rates reaching 70 % (Pappas et al., 2018[31]). The complexity of this scenario and the long periods of hospitalisation result in a financial public health burden (Cortegiani et al., 2017[10]). 

IC may be caused by many *Candida* spp., with *C. albicans* being the most common, followed by other species such as non-*albicans Candida* (NAC) (Colombo et al., 2006[9]; Kullberg and Arendrup, 2015[22]; Antinori et al., 2016[1]; Ostrosky-Zeichner and Al-Obaidi, 2017[30]; Pappas et al., 2018[31]).

Approximately 50 % of IC episodes occur in critically ill individuals treated in intensive care units (ICU). These patients are exposed to a series of factors that favour colonization by *Candida* spp. and, consequently, the risk of IC is increased (Calandra et al., 2016[5]; Gaspar et al., 2015[18]; Leon et al., 2006[24]; Seddiki et al., 2013[37]). Colonization of sites such as the gastric and genitourinary mucosa by yeasts of the *Candida* spp. have been considered an important precondition for subsequent invasive infection in humans (Pappas et al., 2018[31]). The presence of *Candida* spp. in the respiratory tract is a known risk factor for the infection of air passages by multi-resistant microorganisms (Tan et al., 2016[39]); however, little is known about the impact of colonization of the respiratory tract by *Candida *spp., given that the clinical relevance of colonization in the respiratory tract by yeasts is not well established (Eggimann and Pittet, 2014[14], Pendleton et al., 2017[34]).

Biofilms on both biotic and abiotic surfaces have been considered one of the main inoculation sources of microorganisms infecting the lungs (Nett, 2016[29]; Pirrone et al., 2016[35]). The endotracheal tube (ET), besides being an invasive device that puts colonized regions in contact with lung tissue, is also an abiotic surface that facilitates biofilm development. Thus, the use of this device is strongly associated with infections, mostly when used for long periods of time (Danin et al., 2015[11]). However, previous studies have frequently focused on bacterial rather than fungal infections. There is especially a paucity of data from such studies involving yeasts from *Candida *spp., highlighting the lack of knowledge in this respect. Therefore, this study aimed to determine the frequency of colonization by yeasts in the tracheobronchial secretions of intubated patients and to assess the presence of these yeasts in the infra-cuff region of the ET.

## Material and Methods

### Study location and ethics approval

This study was carried out among patients admitted to the adult ICU of the Municipal Hospital of Maringá (MHM) who were under invasive mechanical ventilation (IMV). The study was approved by the Ethics and Research Committee of UniCesumar under the registration number 1.742.787, and family members of all patients provided written informed consent.

### Study population

All patient admissions to the adult ICU from February 2017 to November 2017 were considered for the present study.

The inclusion criteria were as follows: age over 18 years and intubation within the previous 48 hours. Both men and women were included. Based on these criteria, one of the family members was approached and informed about the study, and was asked to sign the informed consent form.

The exclusion criteria were as follows: extubation; death during the time taken for laboratory confirmation of yeast colonization; previous admission to the ICU of another hospital; previous history of tracheostomy; endotracheal intubation for longer than 48 hours; and ET replacement during the period of data collection. Patients were also excluded if they were admitted on a day of the week for which the sample needed to be collected on weekends or holidays. Additionally, we ensured that none of the patients were taking antifungal medications during the study.

### Patient surveillance and isolation and identification of the biological samples

Demographic data obtained from patients included hospital diagnosis at admission, recommendation for mechanical ventilation (MV), administration of antibiotic and antifungal therapy during admission, duration for which the ET was being used, use of a central venous catheter, hemodialysis, and presence of a nasogastric or nasoenteral tube.

The disease severity for each patient during the first 24 hours of admission was evaluated based on the Acute Physiology and Chronic Health Evaluation scale (APACHE II), wherein the variables collected were added to determine a score indicating the probability of death.

Collection of the tracheobronchial secretion was performed during the morning of the day on which the patient would complete 48 hours of intubation. In order to perform this procedure, a sterile flask and suction probe number 14 were used. After collection, a small portion of the biological material was placed in sterile plastic 15-mL conical tubes, containing 3 mL of phosphate buffered saline (PBS, pH 7.5) with antibiotics (polymyxin B and chloramphenicol).

After the patient was extubated, regardless of the reason (weaning of MV, placement of a tracheostomy, or death), the ET was carefully washed with sterile saline solution and, with the aid of a scalpel, the infra-cuff region was cut into two parts. One 2 cm portion was placed in 50 mL plastic conical tubes containing 10 mL of sterile PBS with antibiotics (polymyxin B and chloramphenicol) and dithioerythritol (DTE), and was sent for the isolation and culture of yeasts. The remaining 1 cm fragment was fixed in glutaraldehyde (2.5 %) for 2 hours, and then dehydrated with a graded series of ethanol (70 %, 80 %, 90 %, 95 %, and 100 %). After that, the fragment was treated with gold-palladium and observed under a scanning electron microscope (SEM; Shimadzu SS-550 Superscan, Tokyo, Japan) at the Department of Electronic Microscopy of the State University of Londrina.

At the Medical Mycology division of the Laboratory of Teaching and Research in Clinical Analysis of the State University of Maringá, samples of each tracheobronchial secretion were centrifuged for 5 minutes at 7000 rpm. The supernatant was then discarded, and the pellet was resuspended in 1000 µL of PBS. Afterwards, 10 µL of this suspension was plated in a Petri dish containing Sabouraud dextrose agar (SDA) supplemented with polymyxin B and chloramphenicol, and the dish was incubated at 35ºC for a maximum of 7 days. Then, the number of colony-forming units (CFU) was evaluated and screening was performed in CHROMagar *Candida*^TM^ media to detect the presence of one or more species of Candida based on the color and the characteristics of the colony. This medium was incubated at 35 ºC for 48 hours for final identification. The plaques that did not show any growth in 7 days were considered negative for yeasts.

The 2 cm fragment of the ET was sonicated for 50 seconds at 30 %. Immediately afterward, it was centrifuged for 5 minutes at 7000 rpm. The pellet was resuspended in 1 mL of PBS with antibiotics (polymyxin B and chloramphenicol) and 20 µL was plated on SDA plates following the same procedure detailed above.

Identification of yeasts was performed through classic tests of identification, i.e. by observing micromorphology and using biochemical tests (Kurtzman et al., 2011[23]). This was confirmed using matrix-assisted laser desorption/ionisation-time of flight mass spectrometry (MALDI-TOF MS). Yeast preparation for MALDI-TOF MS was performed according to specific protocols (Spanu et al., 2012[38]; Pascon et al., 2011[33]), and the reading and interpretation were performed using VITEK® MS using Myla® software (bioMérieux, Marcy l'Etoile, France).

### Antifungal susceptibility profile

The antifungal susceptibility profile against amphotericin B (Sigma, Belgium) and fluconazole (Sigma, Belgium) was determined. The test was performed using a microdilution assay in broth, according to the guidelines of the Clinical Laboratory Standards Institute M27-A3 document (CLSI, 2008[8]). Concentrations ranged from 0.125 to 64 µg/ml for fluconazole and between 0.03 and 16 µg/ml for amphotericin B. Suspensions were tested with antifungal solutions in 96-well microplates (Nunclon Delta; Nunc) incubated for 48 hours at 35 ºC. The cell lines *C. albicans* (ATCC 90028) and *C. tropicalis* (ATCC 750) were used as controls. The microplates were read at 405 nm (Expert Plus Microplate Reader; ASYS). The minimum inhibitory concentration (MIC) of amphotericin B was defined as the lowest concentration that completely inhibited growth. The MIC of fluconazole was defined as the lowest concentration that inhibited 50 % of growth. Results were expressed as: susceptible (S), susceptible-dose dependent (S-DD), and resistant (R), with the breakpoints based on the CLSI M60 document (CLSI, 2017[7]). For amphotericin B, resistant isolates were defined as isolates with MIC > 1 µg/ml, as described by Montagna et al. (2014[27]).

### Statistical analysis 

Baseline patient characteristics were compared between patients with and without the presence of *Candida* spp. in the respiratory tract. Categorical variables are described as counts and percentages and were compared using Fisher's exact test. Continuous variables are described as means with standard deviation and were compared using an independent *t*-test. P values < 0.05 were considered statistically significant. Analyses were completed with Statistica version 10 (Statsoft Inc., Tulsa, OK, USA).

## Results

**Figure 1:**
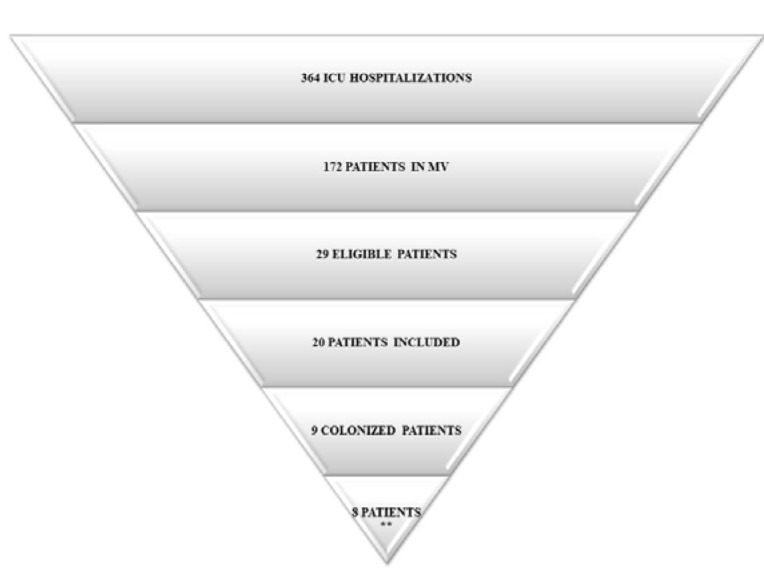
Distribution of patients admitted to the ICU included in the study and representation of the yeasts of *Candida* genus found in the samples. **Eight patients showed colonization by yeasts of the *Candida* genus in both the tracheobronchial secretion and ET. Abbreviations: MV, mechanical ventilation; ICU, intensive care unit; ET, endotracheal tube

Between February and November 2017, 29 patients fulfilled the inclusion criteria for the current study. Of these, 20 patients were included in the data analysis, as there were 2 deaths, 3 patients were transferred to another hospital, 2 patients were extubated before the end of the data analysis, and 2 patients underwent an early tracheostomy. Among the 20 patients, 45 % (n = 9) showed yeast colonization in the respiratory tract; all these yeasts were from *Candida* spp. In 8 of these patients, the same species of yeast was found in the tracheobronchial secretion and in the distal portions of the ET; this group was used for further microbiological analyses (Figure 1(Fig. 1)). It is important to note that one patient (H15) was taking prophylactic antifungal medication at the time of sample collection and was included in the study as an exception because he was HIV+ and his case was thus relevant for microbiological analysis.

The average age of the 20 patients was 73.3 years (± 13.1), and the proportion of males and females was the same. In 55 % of patients (n = 11), the reason for hospitalization involved the lungs. A comparison of these data and other features between patients showing yeast colonization and those not showing yeast colonization is presented in Table 1(Tab. 1).

Among the 9 patients showing yeast colonization in the respiratory tract, 44.4 % (n = 4) were hospitalised for a surgical procedure (exploratory laparotomy) - significantly different from the proportion among patients not showing colonization (*P* = 0.026). In contrast, in the group of patients not showing colonization (n = 11), pulmonary involvement was the main reason (63.6 %) for hospitalisation.

Among the 20 patients, the mean APACHE II score during the first 24 hours of hospitalisation was 29.7 (± 5.3), and the chance of death varied from 40 % to 85 %, without any significant difference between the groups (with or without yeast colonization). In the group without yeast colonization in the respiratory tract, 30 % of patients underwent a tracheostomy. In total, 70 % of all patients (n = 14) died, regardless of colonization by yeasts (*P* = 0.21).

As all patients were under intensive care, a central venous catheter and vesical and nasoenteral probes were used. In 4 patients, a catheter for hemodialysis was also necessary.

Hemoculture and urinoculture were performed during hospitalisation in 80 % and 75 % of the patients, respectively. Candidemia was not diagnosed in any of the patients; however, on urinoculture, 15 % (n = 3) of patients were diagnosed with candiduria caused by NAC species. Of these patients, only one showed colonization in the respiratory tract, albeit by a different species.

On average, the 20 patients remained under endotracheal intubation and MV for 6.4 days (± 1.8) and 13.5 days (± 15), respectively, with no significant difference between the groups. Antibiotic escalation was needed in 65 % of the patients, and there was no significant difference between the two groups in this regard.

As seen in Table 2(Tab. 2), most patients showing yeast colonization in the respiratory tract, except for 1 (H5), showed high counts of yeast infection (> 10^5^ CFU/mL). Moreover, this count was similar in both samples (secretion and ET). In only one patient (P1), although a high count was found in the secretion, the ET count was low. 

All yeasts identified from the culture of tracheobronchial secretions and ET fragments belonged to *Candida *spp., with a predominance of *C. albicans* (66.7 %). The NAC species identified were *C. glabrata* and *C. tropicalis. *In general, isolates of *C. albicans* were obtained as pure cultures, except for in one case (patient H8). In contrast, NAC species were found in co-colonization conditions.

**Figure 2:**
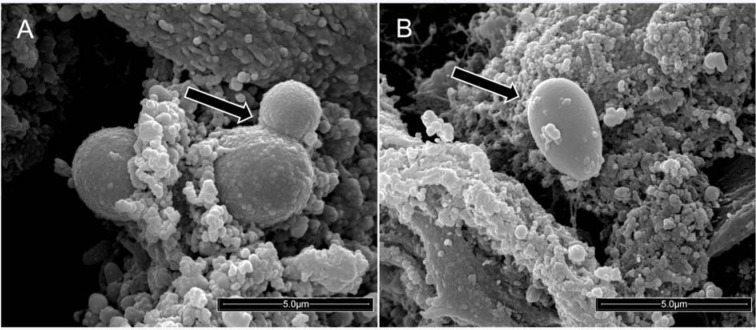
Scanning electron micrograph showing yeasts firmly adhered to the surface of the external portion of the infra-cuff region of a polyurethane endotracheal tube from one extubated patient (20,000× magnification). (A) The arrow indicates a budding yeast. (B) The arrow indicates a yeast in the blastoconidia shape.

Figure 2(Fig. 2) illustrates the presence of these yeasts in the distal portions of the ET. These pictures were taken from a fragment of the ET using scanning electron microscopy (SEM). The distal fragment of the ET after the extubation of one patient was taken *in natura*, and yeasts forming budding cells (Figure 2A(Fig. 2)) or blastoconidia (Figure 2B(Fig. 2)) were observed by SEM. These fungal structures were to be firmly adhered to the ET in a biofilm form, covered by bronchial secretions and surrounded by other microorganisms and extracellular matrix.

Regarding antifungal susceptibility profile, all isolates were susceptible to fluconazole and amphotericin B, irrespective of the species and where they were isolated from (i.e. secretion or ET) (Table 3(Tab. 3)).

## Discussion

In this study, within 48 hours of intubation, 45 % of the patients showed respiratory tract colonization by yeasts of the *Candida *genus*.* Further, in approximately 90 % of these patients, the same yeast was found in both the tracheobronchial secretion and the infra-cuff portion of the ET.

While people including healthy individuals may show *Candida* sp. Colonization without any negative consequences, a simple colonization may evolve into infections that can be localized and cause minor clinical impact in certain groups of patients. In particular, the consequences are more relevant in individuals with extreme health debilities, such as surgical patients (Ha et al., 2011[21]) or older patients with other morbidities (Guimarães et al., 2012[20]). Moreover, patients who need endotracheal intubation, MV, ICU hospitalization for more than a week, and catheterization show considerable chances of this same colonization evolving to IC (Muskett et al., 2011[28]), which is associated with high mortality and morbidity.

Despite their severity, there are several limitations to the final diagnosis of invasive infections (Eggiman et al., 2015[15]). Hemocultures provide positive results in only 50-70 % of cases (Calandra et al., 2016[5]). Considering that bacterial infections have historically received more attention than the fungal ones, preventive prescription and escalation of broad-spectrum antibiotics is common. Thus, antibiotic use represents an additional risk factor for the occurrence of invasive fungal infections (Delaloye and Calandra, 2014[12]; Eggiman et al., 2015[15]; Muskett et al., 2011[28]) due to the selective pressure on the pathogenic microorganisms.

*Candida albicans* was the most frequently found species among all the isolates (66.7 %) in our study. These data are related to colonization, but this species is also responsible for 38-70 % of IC cases (Colombo et al., 2006[9]; Kullberg and Arendrup, 2015[22]; Antinori et al., 2016[1]; Ostrosky-Zeichner and Al-Obaidi, 2017[30]; Pappas et al., 2018[31]). This frequency varies based on patient characteristics, the state of the disease, and the geographic region. Our findings of *C. glabrata* and *C. tropicalis* in patient samples are also consistent with infections caused by NAC, as these species are among the five most frequently isolated and reported worldwide.

Tracheobronchial secretions contain various microorganisms in planktonic form, usually with minor clinical impact. However, the surface of the ET favors adherence of these bacteria and fungi, which may organize themselves as a biofilm (Ferreira et al., 2016[17]). As shown in Figure 1(Fig. 1), most patients with yeasts present in the tracheobronchial secretion also showed yeast colonization in the distal portions of the ET (infra-cuff regions). Additionally, as shown in Table 2(Tab. 2), the same species were found in both samples. In a recent study, Ferreira et al. (2016[17]) did not find differences between the microorganisms isolated from the tracheal aspirate and the extubated ET. It is important to highlight that the methodological protocol chosen in the present study was standardized during a pilot study performed previously. Thus, analyzing the yeasts in pure cultures was only possible after washing the ET in order to remove the microorganisms not adhered to it. Then, following sonication, the yeasts that were firmly adhered to the ET were cultivated in selective medium (SDA) and treated with a wide range of antibiotics to provide pure cultures of yeasts.

As shown in Table 3(Tab. 3), there were no differences in the responses of yeasts to antifungal drugs: yeasts isolated both from the ET and the tracheobronchial secretion were susceptible to amphotericin B and fluconazole. Thus, yeasts disseminated throughout the secretion were likely the same as those found in the ET.

Interestingly, all isolates were sensitive to the tested antifungals, which are the most frequently used agents in the hospital where the study took place. Yeasts susceptible to amphotericin B and isolated from ETs have already been reported by other authors (Modrezewska et al., 2017[26]; Baghdadi et al., 2016[3]). However, these same authors found a relatively low rate of resistance to fluconazole in these yeasts. Badiee et al. (2017[2]) have also found differences in the susceptibility of *Candida* spp. to antifungals depending on whether the yeasts were obtained from the microbiota or from an infection, mainly in immunocompromised patients.

Scanning electron micrographs highlighted that the yeasts in biofilms, in blastoconidia shape, strongly adhered to the surface of the ET removed from a patient (Figure 2A and B(Fig. 2)). Indeed, the results lead to the conclusion that *Candida* spp. show a high capacity to form biofilms on abiotic surfaces such as the ET. In this condition, the yeasts lie in a matrix of polysaccharides, proteins, and other components that protect the yeast against the host's immune system, as well as grant the yeasts resistance to antibiotics (Douglas, 2003[13]; Chandra et al., 2001[6]). It is important to highlight that this biofilm structure formed by the yeasts occurred naturally on the ET surface inserted in the respiratory tract. The biofilm works as a reservoir for these microorganisms, representing an important etiological risk factor for pulmonary infections, due to the dispersion effect that occurs in parts of the biofilm, allowing translocation of these agents to the lower airways (Fernández-Barat et al., 2012[16]; Gil-Perotin et al., 2012[19]; Bassi et al., 2015[4]; Meligy et al., 2015[25]). Importantly, yeasts detached from the biofilm have an increased capacity of invasion (Uppuluri et al., 2018[40]) and thus pose a real risk of pulmonary infection.

There is no consensus yet on the relevance of laboratory findings of yeasts in the respiratory tract and their relationship with pulmonary complications (Ricard and Roux, 2012[36]). However, there is evidence that *Candida* spp. may be an offensive agent in ventilated patients hospitalized in the ICU, as this procedure can facilitate clinical worsening. Thus, the role of the fungal biofilm, possibly in combination with bacteria, should not be neglected (Pascale and Antonelli, 2014[32]).

Even though we did not register any cases of IC in the patients of this study and the high death rate could not be associated with the colonization found, our data provide important insights. Interestingly, our study included critically ill patients, and the results reinforce the idea that it is still challenging to identify individuals who may benefit from antifungal prophylaxis and empirical treatment. Patients with yeast colonization are usually exposed to many risk factors, and the fact that yeasts were found in the infra-cuff regions of the ET suggests the multiplication of these predisposing factors. 

Our study had a few limitations. Owing to the short follow-up period, delineation aimed at obtaining data regarding colonization during the first hours of intubation was one limitation. Possibly, observation over a longer period may provide a better correlation between colonization and outcome. A longer duration of MV could also affect the data and possibly lead to the observation of a greater frequency of colonization, as well as a greater potential for the development of IC. However, we also have to consider that the approach taken in the management of this type of patients is dynamic, and the conditions of critically ill patients may complicate the feasibility of a longer follow-up. For instance, some of the patients required tracheostomy and many of them died; there were also other factors making it difficult to follow patients over a longer period. Another limitation of our study was the small sample size. However, our results serve as the first step in providing information on this medical issue. Further research is warranted to identify yeasts in secretions and in the ET, and to characterize the biofilms. Future studies could help in validating the presence of yeasts in the tracheobronchial secretion as an essential surveillance parameter among critically ill patients and help minimize the risks of IC.

## Conclusion

The results of the present study demonstrated that the frequency of colonization by yeasts in the tracheobronchial secretions of patients intubated for 48 hours is high. Moreover, we found that the yeasts disseminated in the secretions are similar to the ones found in the infra-cuff region of the ET with regard to the species, number of microorganisms, and antifungal susceptibility profile. Therefore, such colonization deserves attention, as it can be an additional important risk factor for pulmonary infections.

## Acknowledgements

This study was supported by Coordenação de Aperfeiçoamento de Pessoal de Nível Superior (CAPES), Conselho Nacional de Desenvolvimento Científico e Tecnológico (CNPq), Fundação de Amparo à Pesquisa do Estado do Paraná (Fundação Araucária), and Laboratory of Electron Microscopy and Microanalysis, Universidade Estadual de Londrina, Londrina, Paraná, Brazil.

## Declaration of conflicting interests

The authors declare no potential conflicts of interest with respect to the research, authorship, and/or publication of this article.

## Figures and Tables

**Table 1:**
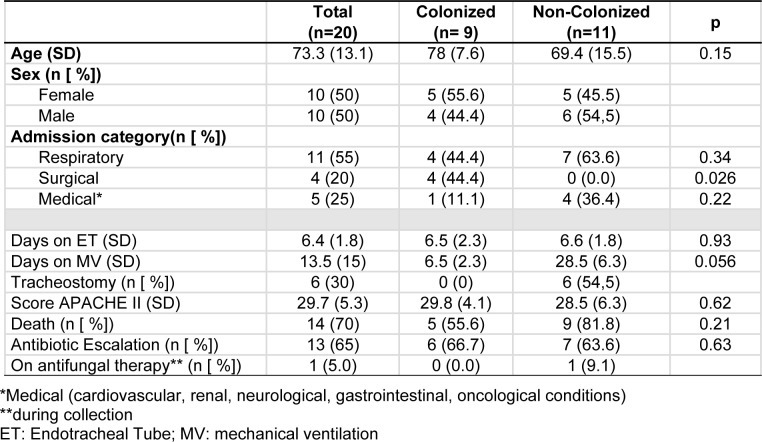
Patient details

**Table 2:**
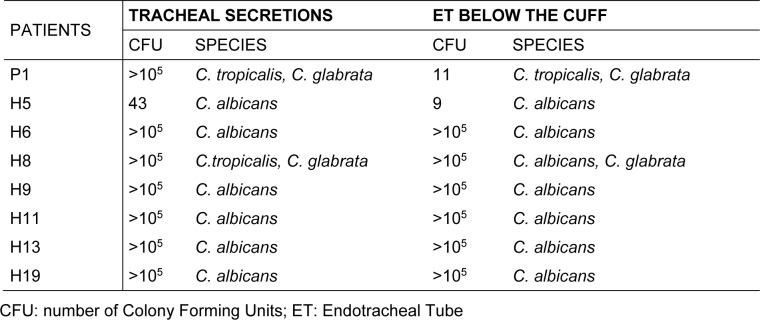
Distribution by species and count of colony forming units of the yeasts obtained from the eight colonized patients concomitantly in the tracheobronchial secretion and also in the ET

**Table 3:**
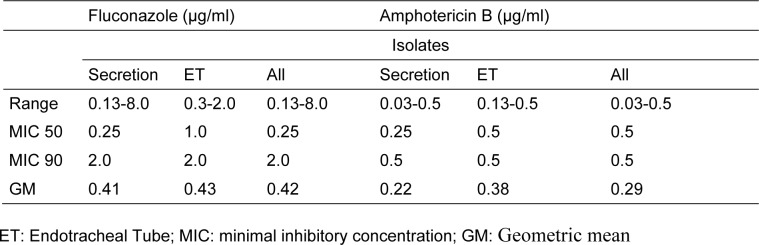
Antifungal susceptibility profile of yeasts isolated and identified from colonized patients in both tracheobronchial secretion and ET
